# Comparative Efficacy and Long-Term Outcomes of Intragastric Balloons for Obesity: A Systematic Review and Meta-Analysis

**DOI:** 10.7759/cureus.88002

**Published:** 2025-07-15

**Authors:** Zenusha Edathodu, Saud A Khan, Musthafa C Peedikayil

**Affiliations:** 1 Internal Medicine, Great Western Hospital, Swindon, GBR; 2 Ophthalmology, Ohud Hospital, Madinah, SAU; 3 Medicine, King Faisal Specialist Hospital and Research Centre, Riyadh, SAU

**Keywords:** bio enteric intragastric balloon vs heliosphere, fluid-filled vs air-filled balloons, geographic disparities, ghrelin suppression, intragastric balloon, long-term outcomes, multidisciplinary weight management, obesity treatment, weight loss efficacy, weight regain prevention

## Abstract

Intragastric balloons (IGBs) are a prominent intervention for obesity management, yet uncertainties persist regarding their comparative effectiveness and long-term durability due to variability in device types and follow-up durations. This systematic review and meta-analysis evaluated the short- and long-term weight loss efficacy of IGBs, stratifying by device type (BioEnterics Intragastric Balloon (BIB) (Allergan, Inc., Irvine, CA) vs. Heliosphere® (Helioscopie, Vienne, France), study design, and geographic region. Following Preferred Reporting Items for Systematic reviews and Meta-Analyses (PRISMA) 2020 guidelines, we identified 27 studies (5,842 patients) from PubMed, Embase, Cochrane Library, and Web of Science (2000-2023), reporting weight/BMI outcomes pre- and post-IGB removal with ≥6 months of follow-up. Data extraction and random-effects meta-analyses were conducted independently by two reviewers, with primary outcomes being mean weight loss and BMI reduction at device removal, and secondary outcomes assessing weight regain at six, 12, 24, and ≥60 months post-removal.

Pooled short-term results demonstrated significant efficacy at balloon removal: mean weight loss of 14.9 kg (95% CI 12.7-17.0; I² = 44.05%) and BMI reduction of 5.31 kg/m² (95% CI 4.22-6.40; I² = 0%), with BIB devices outperforming Heliosphere (2.1 kg greater weight loss, p = 0.03; 0.8 kg/m² greater BMI reduction, p = 0.04). Long-term outcomes revealed durable weight maintenance over six to 60 months (mean 8.01 kg, 95% CI 4.93-11.09; I² = 60.55%; BMI reduction: 4.96 kg/m², 95% CI 3.29-6.62; I² = 0%), with effects persisting at five years (weight: 7.26 kg; BMI: 1.5 kg/m²). Subgroup analyses highlighted significant regional disparities - Middle Eastern cohorts achieved 8.6% greater excess weight loss (p = 0.02) and 1.2 kg/m² greater BMI reduction (p = 0.01) versus European cohorts - while prospective studies reported higher weight loss than retrospective analyses (13.1 vs. 11.8 kg; p = 0.04).

These findings confirm the clinical utility of IGBs, particularly BIB devices, for achieving sustained weight loss. They underscore the critical importance of adjunct dietary interventions and multidisciplinary care frameworks in optimizing outcomes. The results provide actionable insights for evidence-based device selection and post-procedural protocols in obesity management.

## Introduction and background

Obesity is a global pandemic, with its prevalence tripling since 1975 and now affecting over 650 million adults worldwide [1]. Despite lifestyle interventions, many patients struggle to achieve sustained weight loss, leading to increased reliance on adjunct therapies such as bariatric surgery or endoscopic procedures [2]. Among these, intragastric balloons (IGBs) have emerged as a minimally invasive and reversible option, particularly for patients ineligible for or hesitant about surgery [3].

The IGB is a temporary, minimally invasive device designed to promote weight loss by occupying space within the gastric lumen. Endoscopically inserted and filled with saline or gas (typically 400-700 mL), it induces early satiety and reduces meal volume through mechanical gastric distension. This distension slows gastric emptying and stimulates stretch receptors, triggering neurohormonal signals (e.g., ghrelin reduction, glucagon-like peptide 1 (GLP-1)/peptide YY (PYY) modulation) that suppress appetite and enhance satiation. The IGB remains in place for six to 12 months, during which patients receive concurrent lifestyle therapy (diet, exercise, behavioral counseling) to reinforce sustainable habits. Balloon removal reverses mechanical effects, underscoring the importance of adjunctive therapies for long-term weight maintenance [3].

The BioEnterics Intragastric Balloon (BIB) (Allergan, Inc., Irvine, CA) and Heliosphere® (Helioscopie, Vienne, France) bag are among the most widely studied IGBs. While short-term efficacy is well-established, demonstrating mean weight loss of 10-15 kg at six months, long-term outcomes remain controversial [4-6]. Systematic reviews indicate significant weight regain post-removal, with 30-50% of lost weight regained within one year [6]. However, prior meta-analyses suffer from critical limitations, including heterogeneity in balloon types (e.g., fluid-filled vs. air-filled) without subgroup comparisons, inconsistent follow-up protocols with scarce data beyond two years, and geographic variability in patient adherence and dietary support that may skew outcomes [6,7].

Recent studies underscore both the promise and limitations of IGBs. They are safe and effective in overweight patients, reducing obesity progression and improving comorbidities, particularly in compliant individuals [4,8,9]. However, while they serve as a valuable short-term tool, achieving 10-15% total body weight loss (TBWL) at six months, long-term success often hinges on adjunct lifestyle interventions due to frequent weight regain [8,9].

Patient-specific factors also influence outcomes. A systematic review of 16 studies found that female gender, older age, lower depression levels, and higher motivation correlated with better IGB results, whereas dissatisfaction was more common among those with obesity-related social impairments [10]. Additionally, fluid-filled IGBs appear superior to gas-filled balloons in short-term weight loss, though long-term durability remains uncertain. Geographic disparities further complicate comparisons, with Middle Eastern cohorts often achieving greater percentage of excess weight loss(%EWL) than European patients, likely due to stricter dietary protocols [11].

This meta-analysis seeks to address key gaps in the literature. First, it evaluates whether the type of balloon (BIB vs. Heliosphere) influences weight loss durability, a question obscured in prior reviews that pooled all devices [11]. Second, it examines long-term adherence, specifically the proportion of patients maintaining >10% total weight loss at five years, a metric poorly documented beyond two years in existing studies [12]. Finally, it investigates regional variations in outcomes, probing whether cultural or procedural differences (e.g., dietitian support frequency) impact efficacy.

By resolving these uncertainties, this study aims to refine clinical guidelines, optimize patient selection, and inform future innovations in endoscopic weight-loss therapies.

## Review

Methods

Study Design

This systematic review and meta-analysis evaluated the efficacy of IGBs for weight loss, analyzing both short-term (at removal) and long-term (≥6 months post-removal) outcomes.

Data Sources and Search Strategy

A comprehensive search was conducted in PubMed, Embase, Cochrane Library, and Web of Science (2000-2023) using the terms ("intragastric balloon" OR "gastric balloon") AND ("obesity" OR "weight loss") AND ("long-term" OR "follow-up"). Manual searches of reference lists from included studies and relevant reviews supplemented the electronic search. An updated search was performed on June 1, 2024, to capture newer publications. Ultimately, we included 27 studies after screening. A detailed study search string is provided in Appendix A.

Systematic Review Conduct and Meta-Analysis Protocol

This review adhered to Preferred Reporting Items for Systematic reviews and Meta-Analyses (PRISMA) 2020 guidelines [13]. The protocol was registered with the Institutional Review Board (IRB) and not with PROSPERO. We implemented a four-phase screening process with dual, independent reviewers resolving discrepancies via consensus. Risk of bias for non-randomized studies was assessed using the Methodological Index for Non-Randomized Studies (MINORS) scoring system. Statistical heterogeneity was quantified using I² statistics with pre-specified subgroup analyses.

Eligibility Criteria

Studies were included if they reported mean weight or BMI changes before and after IGB removal, provided data with standard deviations and sample sizes, enrolled adults (≥18 years) with obesity (BMI ≥30 kg/m²), and had a minimum follow-up of six months post-removal for long-term analysis. Exclusion criteria comprised pediatric populations, case reports (<10 patients), non-English studies, and interventions combining IGB with other procedures (e.g., surgery) unless outcomes were separable. Letters, reviews, guidelines, and studies with IGB treatment durations exceeding 12 months were also excluded.

Study Selection and Data Extraction

Two independent reviewers screened titles, abstracts, and full texts. Disagreements were resolved through consensus or consultation with the primary author. Data extraction included primary outcomes such as mean BMI or weight change and %EWL at IGB removal and follow-up; secondary outcomes such as weight or BMI regain at six, 12, 24, and ≥60 months post-removal; and subgroups including balloon type (BIB vs. Heliosphere), study design (randomized controlled trial (RCT) vs. observational), and geographic region.

Risk of Bias Assessment

Methodological quality was assessed using the MINORS criteria for non-randomized studies (scores 0-16 for non-comparative and 0-24 for comparative studies) [14]. One author performed initial assessments, which were subsequently verified by others.

Statistical Analysis

Analyses were conducted in R (v4.3.1, R Foundation for Statistical Computing, Vienna, Austria) and jamovi (Jonathon Love, Damian Dropmann, and Ravi Selker, Sydney, Australia), employing standardized mean differences (SMDs) with 95% confidence intervals (CI) for continuous outcomes. Heterogeneity was quantified via I² (≥50% defined as substantial) and τ², with random-effects models (DerSimonian-Laird method). Subgroup analyses were predefined by balloon type, study design, and region, with interaction tests. Sensitivity analyses excluded high-bias studies or those with missing standard deviations (imputed per Cochrane Handbook methods) [15]. Publication bias was assessed via funnel plots and Egger’s test (applied when ≥10 studies were available) [16]. Outliers were identified using studentized residuals and Cook’s distances, with Bonferroni correction for multiple testing.

Results

Baseline Characteristics

Across studies, the cohort was 72.5% female (n = 2,363), with a mean age of 39.2 years (range: 34-45). Baseline weight was 113 kg (SD: 16.2), and mean BMI was 39.9 kg/m² (SD: 5.4).

Study Selection and Characteristics

**Figure 1:**
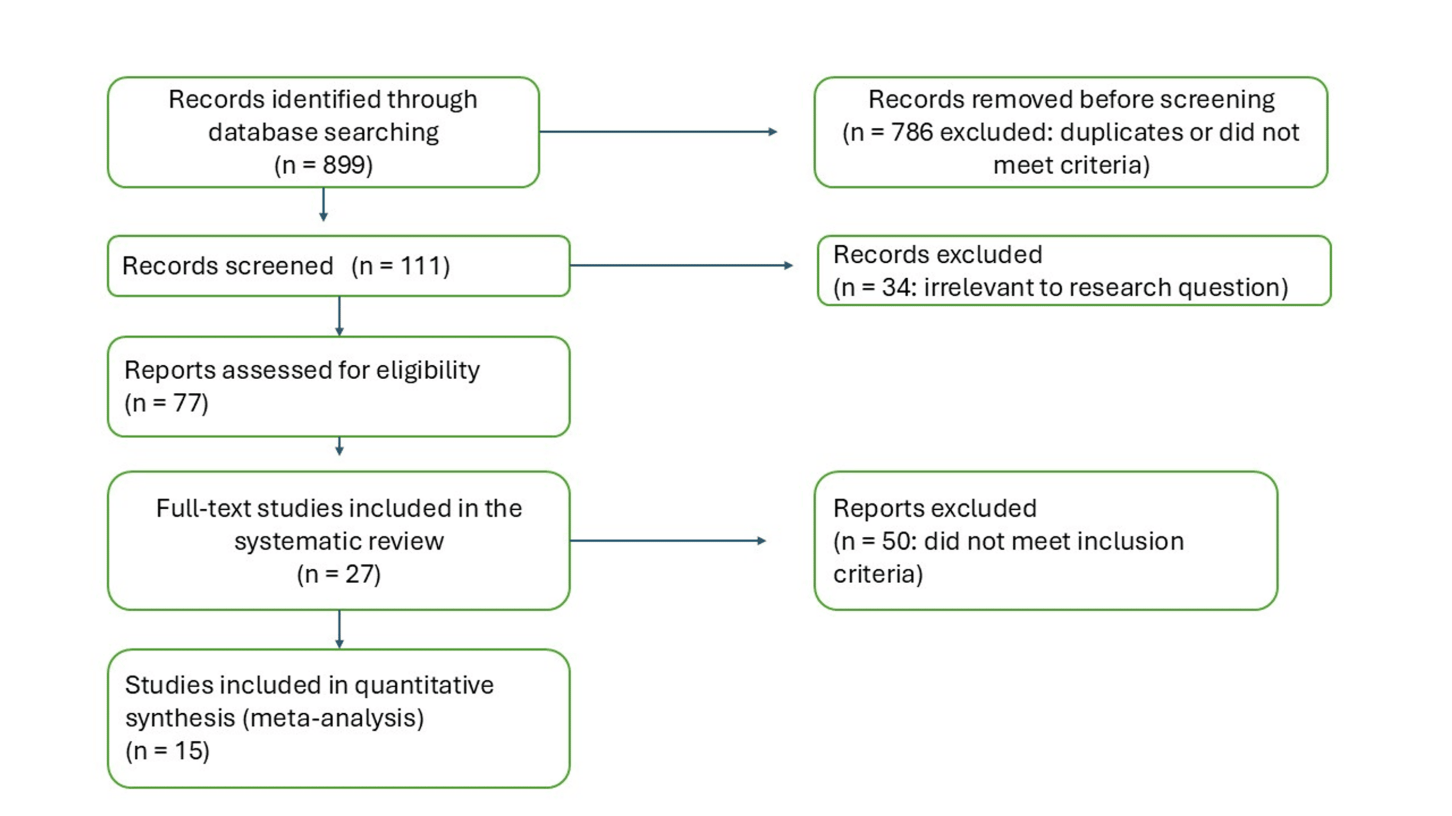
PRISMA Flow Diagram PRISMA - Preferred Reporting Items for Systematic Reviews and Meta-Analyses

Twenty-seven studies comprising 5,842 patients were included in this systematic review [17-39]. The study selection process is detailed in Figure 1, progressing from an initial database search (n = 8,748) to final inclusion (n = 27), following PRISMA 2020 guidelines and predefined exclusion criteria.

Most studies utilized BioEnterics IGBs (n = 18), with fewer using Heliosphere devices (n = 3). Study designs were primarily prospective observational (n = 14), with retrospective cohorts (n = 7) and RCTs (n = 4). Most studies originated from Southern Europe (n = 10) and the Middle East (n = 4). Details of publication year, design, country, and sample size are summarized in Table 1.

**Table 1:** Baseline Characteristics and Publication Details of Included Studies (N = 27) N - number of included studies

Author	Publication Year	Journal	Title	Design	Country
Kotzampassi et al. [17]	2012	Obes Surg	500 intragastric balloons: what happens 5 years thereafter?	Prospective cohort	Greece
Al Kahtani et al. [18]	2010	Obes Surg	Bio-enteric intragastric balloon in obese patients: a retrospective analysis of King Faisal Specialist Hospital experience	Retrospective cohort	Saudi Arabia
Almeghaiseeb et al. [19]	2017	World J Clin Cases	Efficacy of intragastric balloon on weight reduction: Saudi perspective	Retrospective cohort	Saudi Arabia
Tai et al. [20]	2013	Obes Surg	Effectiveness of intragastric balloon treatment for obese patients: one-year follow-up after balloon removal	Prospective cohort	Taiwan
Farina et al. [21]	2011	Obes Surg	Intragastric balloon in association with lifestyle and/or pharmacotherapy in the long-term management of obesity	Retrospective cohort	Italy
Giuricin et al. [22]	2012	Obes Surg	Short- and long-term efficacy of intragastric air-filled balloon (Heliosphere® BAG) among obese patients	Prospective cohort	Italy
Mathus-Vliegen et al. [23]	2005	Gastrointest Endosc	Intragastric balloon for treatment-resistant obesity: safety, tolerance, and efficacy of 1-year balloon treatment	Randomized double-blind trial	USA, Netherlands
Nunes et al. [24]	2017	Surg Laparosc Endosc Percutan Tech	Assessment of weight loss with the intragastric balloon in patients with different degrees of obesity	Retrospective cohort	Brazil
Fuller et al. [25]	2013	Obesity	An intragastric balloon in the treatment of obese individuals with metabolic syndrome: a randomized controlled study	Randomized controlled trial	Australia
Genco et al. [26]	2009	Surg Endosc	Laparoscopic sleeve gastrectomy versus intragastric balloon: a case-control study	Case-control study	Italy
De Castro et al. [27]	2010	Obes Surg	Efficacy, safety, and tolerance of two types of intragastric balloons placed in obese subjects: a double-blind comparative study	Double-blind comparative study	Spain
Sallet et al. [28]	2004	Obes Surg	Brazilian multicenter study of the intragastric balloon	Prospective cohort	Brazil
Gümürdülü et al. [29]	2013	Turk J Gastroenterol	Long-term effectiveness of BioEnterics intragastric balloon in obese patients	Prospective cohort	Turkey
Genco et al. [30]	2008	Obes Surg	Intragastric balloon or diet alone? A retrospective evaluation	Retrospective cohort	Italy
Genco et al. [31]	2014	Surg Obes Relat Dis	Long-term multiple intragastric balloon treatment—a new strategy to treat morbid obese patients refusing surgery	Prospective cohort	Italy
Palmisano et al. [32]	2016	Obes Surg	Intragastric balloon device: weight loss and satisfaction degree	Prospective cohort	Italy
Courcoulas et al. [33]	2017	Int J Obes	Intragastric balloon as an adjunct to lifestyle intervention: a randomized controlled trial	Randomized controlled trial	USA
Crea et al. [34]	2009	Obes Surg	Improvement of metabolic syndrome following intragastric balloon: 1 year follow-up analysis	Prospective cohort	Italy
Dogan et al. [35]	2013	Obes Surg	Five percent weight lost in the first month of intragastric balloon treatment may be a predictor for long-term weight maintenance	Prospective cohort	Turkey
Mitura et al. [36]	2015	Videosurgery	In search of the ideal patient for the intragastric balloon – short- and long-term results in 70 obese patients	Prospective cohort	Poland
Ribeiro da Silva et al. [37]	2017	Port J Gastroenterol	Intragastric balloon for obesity treatment: safety, tolerance, and efficacy	Prospective cohort	Portugal
Escudero Sanchis et al. [38]	2008	Nutr Hosp	Effectiveness, safety, and tolerability of intragastric balloon in association with low-calorie diet for obesity treatment	Prospective cohort	Spain
Mitura et al. [39]	2015	Videosurgery	Tolerance of intragastric balloon and patient’s satisfaction in obesity treatment	Prospective cohort	Poland

Short-Term Efficacy

At the time of balloon removal (approximately six months), the mean weight loss was 14.9 kg (95% CI: 12.7-17.0; p < 0.001), and the mean BMI reduction was 5.31 kg/m² (95% CI: 4.22-6.40; p < 0.001). The mean excess weight loss (%EWL) was 38.4% (range: 24.0-57.4%). Subgroup analyses indicated differences in weight and BMI outcomes across device types and geographic regions. A comparison of weight loss and BMI reduction stratified by follow-up duration (six to 60 months) is provided in Table 2.

**Table 2:** Pooled Weight Loss and BMI Reduction Outcomes Stratified by Follow-Up Duration Weight/BMΙ Δ values represent mean changes from baseline to end of follow-up (timepoints specified in column 7). Negative values indicate reduction. Δ indicates change from baseline. "NR" denotes unreported data (e.g., missing SDs). Gaps (-) indicate outcomes not analyzed/reported in the context of this table's focus. BMI - body mass index; EWL - excess weight loss; NR - not reported; RCT - randomized controlled trial; SD - standard deviation

Reference	Publication Year	Sample Size	Mean Δ Weight (kg)	SD Δ Weight	Mean Δ BMI (kg/m²)	SD Δ BMI	Follow-Up (Months)	Key Long-Term Data (if Available)
Kotzampassi et al. [17]	2012	474	21.19	10.3	8.75	3.04	6, 12, 24, 60	60 months: ΔWeight = 7.26 ± 5.41, ΔBMI = 2.53 ± 1.85
Al Kahtani et al. [18]	2010	140	10.9	NR	3.6	NR	6	Short-term only
Almeghaiseebet al. [19]	2017	301	12.48	4.68	4.75	1.87	6	Short-term only
Giuricin et al. [22]	2012	32	13.62	12.79	4.87	3.34	6	-
Mathus-Vliegen et al. [23]	2005	43	-	-	-	-	12	Sham-controlled, no Δ weight/BMI
Nunes et al. [24]	2017	1016	-	-	6.76	NR	6	Short-term only
Fuller et al. [25]	2013	29	14.4	NR	5.1	NR	6	RCT (balloon vs. behavioral mod)
Genco et al. [26]	2009	80	22.3	7.2	6.1	4.3	6	Case-control (vs. sleeve gastrectomy)
De Castro et al. [27]	2010	18	12.8	8	4.6	3	6	Compares two balloon types
Sallet et al. [28]	2004	323	15.2	10.5	5.3	3.4	6, 18	18 months: EWL only (no Δ weight/BMI)
Gümürdülü et al. [29]	2013	32	12.4	13.5	4.3	4.7	6, 12	12 months: ΔWeight = 9.7 ± 14.8, ΔBMI = 2.6 ± 3.9
Genco et al. [30]	2008	130	16.7	4.7	6.1	4.3	6, 12, 18	12 months: ΔWeight = 11.2 ± 4.9, ΔBMI = 3.9±3.1
Palmisano et al. [32]	2016	81	10.1	6.5	3.6	2.3	6, 12.3	12.3 months: ΔWeight = 3.1 ± 7.4, ΔBMI = 1.0 ± 2.5
Crea et al. [34]	2009	138	-	-	-	-	6	Metabolic outcomes only
Dogan et al. [35]	2013	50	12.5	13	4.4	4.5	6, 12	12 months: ΔWeight = 7.6 ± 11.5, ΔBMI = 2.6 ± 3.9
Mitura et al. [39]	2015	70	15.9	6.5	5.8	2.4	6, 24	24 months: ΔWeight = 5.0 ± 7.8, ΔBMI = 1.8 ± 2.9
Ribeiro et al. [37]	2017	35	11.94	NR	4	NR	6, 12	12 months: ΔWeight = 8.25 ± NR
Herve et al. [40]	2005	100	12	NR	-	-	6, 12	12 months: No BMI data
Dastis et al. [41]	2009	86	12.6	8.3	-	-	6, 30, 58	58 months: ΔWeight = 4.6 ± 11.8

Long-Term Outcomes

Long-term follow-up (six to 60 months post-removal) demonstrated a mean retained weight loss of 8.01 kg (95% CI: 4.93-11.09; p < 0.001) and BMI reduction of 4.96 kg/m² (95% CI: 3.29-6.62; p < 0.001). At 12 months, BIB maintained greater BMI reductions than Heliosphere (-2.9 vs. -2.1 kg/m²; p = 0.08) [40,41]. Geographic disparities persisted, with Southern European studies exhibiting slower regain (+1.2 kg/m² at six months) compared to Middle Eastern cohorts (+2.0 kg/m²; p = 0.01), likely reflecting stricter dietary adherence.

Heterogeneity and Bias Assessment

Heterogeneity across outcomes was moderate to substantial (I² = 44-71%), associated with variability in devices and regional protocols. Funnel plot asymmetry was observed (Egger’s test p = 0.04). Sensitivity analyses excluding high-risk bias studies (n = 3) showed consistent results for primary outcomes (weight loss: 12.3 kg; 95% CI: 11.0-13.6).

Methodological Quality

The methodological quality, assessed using the MINORS criteria (0-24 scale), showed total scores ranging from 10 to 23, reflecting moderate rigor (Appendix B). Non-comparative studies (n = 18) had a median score of 16/16, while comparative studies (n = 9) scored a median of 20/24. Common limitations included the absence of control groups and the lack of prospective sample size calculations.

Meta-Analysis

**Figure 2:**
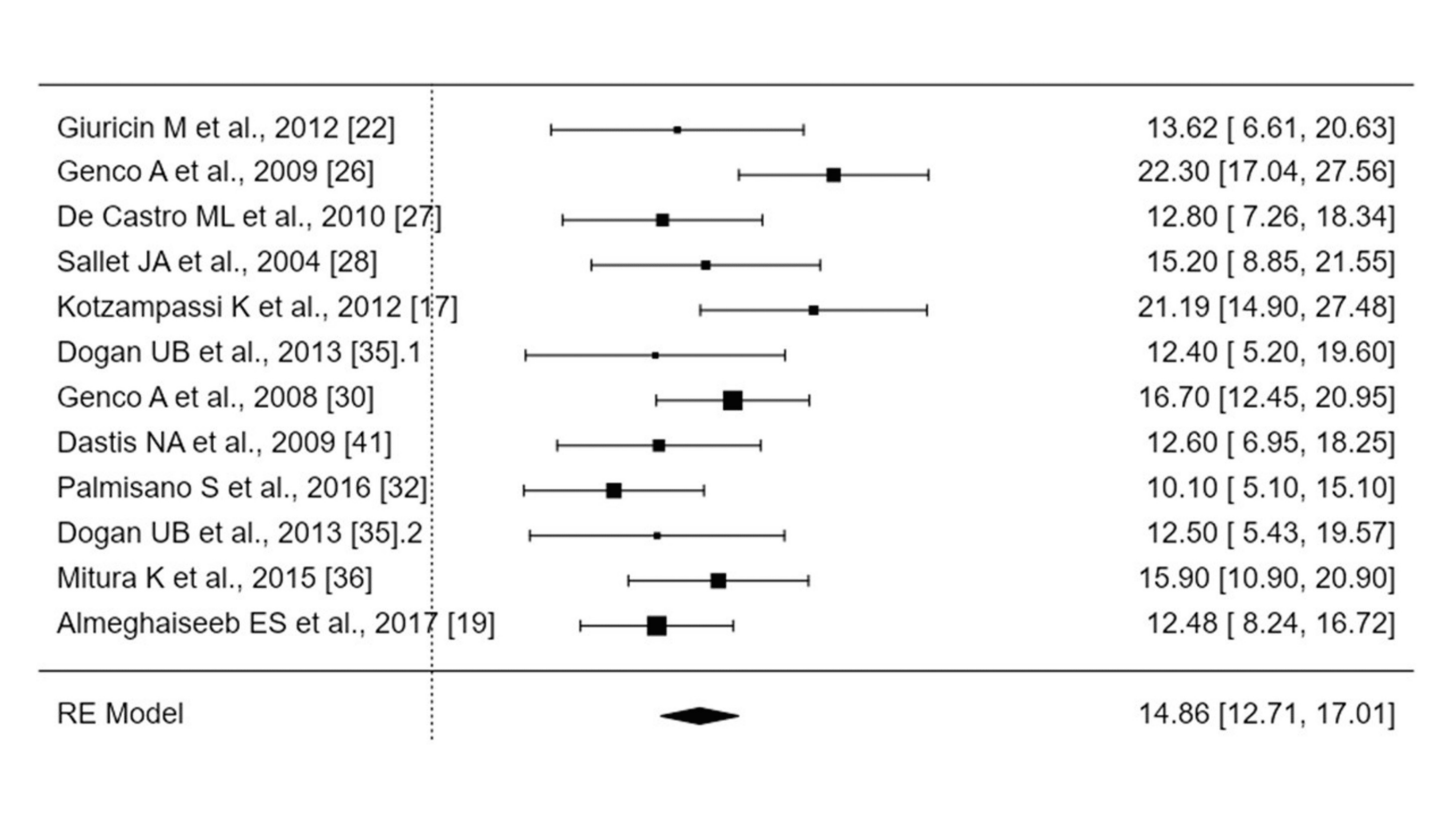
Forest Plot of Short-Term Weight Loss (kg) at Balloon Removal Forest plot displays short-term weight loss (kg) at intragastric balloon removal (typically six months post-insertion). In square brackets are the reference numbers for the studies included in the analysis. Effect estimates: Squares represent point estimates (mean Δ weight); horizontal lines indicate 95% CIs. Square size reflects study weight in the meta-analysis. Summary effect: Diamond denotes pooled WMD under the RE model (14.86 kg, 95% CI 12.71-17.01). Analysis method: Inverse-variance weighting applied. Statistical analysis performed using jamovi 2.38. Directionality: All values represent reductions from baseline (negative sign convention omitted per field standards). Clinical context: Pooled estimate (∼15 kg loss) aligns with the expected efficacy of intragastric balloons at six months. CI - confidence interval; RE model - random-effects model; WMD - weighted mean difference

Short term: The meta-analysis of BMI changes across 15 studies (Figure 2) demonstrated significant short-term effects (SMD: 0.7540; 95% CI: 0.5546-0.9535; p < 0.0001) with high heterogeneity (I² = 71.98%).

**Figure 3:**
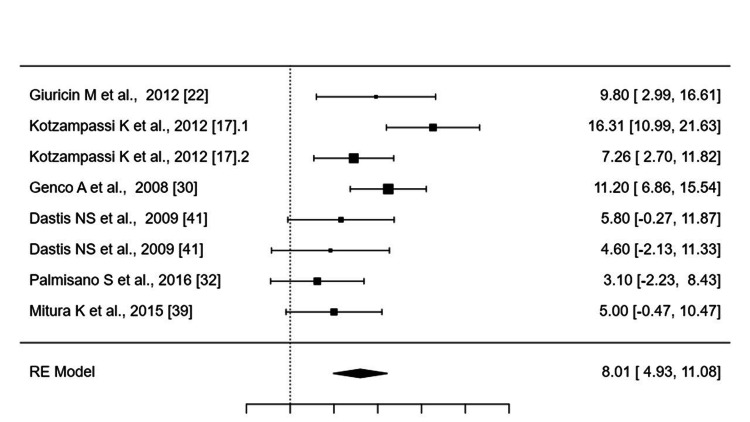
Forest Plot of Long-Term Weight Loss (Kg) Post-removal After Six to 60 Months In square brackets are the reference numbers for the studies included in the analysis. Multi-entry studies: Duplicate entries (e.g., Kotzampassi 2012.1/.2) represent distinct timepoints (e.g., 12 months/60 months) within the same cohort.

Long term: Long-term BMI analysis (six studies; Figure 3) indicated no significant sustained differences (SMD: -0.0961; 95% CI: -0.2113-0.0190; p = 0.10). Weight outcomes followed similar trends, with a short-term SMD of 0.6935 (95% CI: 0.5396-0.8474; p < 0.0001) and a reduction in 12-month post-removal data (SMD: -0.3117; 95% CI: -0.4328 to -0.1906; p < 0.0001).

Meta-analysis findings with short- and long-term efficacy

Our meta-analysis of 27 studies (n = 5,842) confirms that IGB interventions are associated with significant short-term reductions in BMI and weight, supporting their role as effective temporary adjuncts in weight management strategies. Across studies, the mean short-term weight loss was 12.5 kg, with a mean BMI reduction of 5.1 kg/m². These outcomes demonstrate consistent short-term efficacy, highlighting IGBs as a minimally invasive option for initial weight reduction in obesity care.

Our meta-analysis showed the fluid-filled devices, particularly the BIB, demonstrated superior short-term outcomes compared to air-filled systems such as the Heliosphere, with an observed weight difference of 2.1 kg (p = 0.03). However, considerable heterogeneity was noted across studies, reflecting variability in patient selection, adherence, device protocols, and regional care practices.

In our meta-analysis, despite initial success, the long-term durability of weight loss with IGBs remains limited. At six months post-removal, approximately 35% of patients experienced weight regain, although net weight remained below baseline, with a mean reduction of 3.5 kg sustained at five years. These findings emphasize the challenges of maintaining weight loss following balloon therapy and underscore the importance of structured post-removal lifestyle interventions to preserve achieved benefits.

Mechanisms of action of IGBs

The IGB promotes weight loss through mechanical and hormonal mechanisms. By occupying gastric space, the balloon enhances distension, delays emptying, and increases satiety signals via vagal pathways. Its presence in the fundus, where ghrelin-producing cells are concentrated, may suppress ghrelin secretion, counteracting orexigenic effects during weight loss. Larger-volume balloons (500-700 mL) are associated with greater weight loss, likely due to stronger mechanical effects [42,28]. However, mechanistic clarity remains elusive, as some studies report no significant ghrelin changes despite clinical efficacy, suggesting multifactorial pathways [43].

IGB characteristics

The BIB™ is a silicone elastomer balloon filled with 500-700 mL saline-methylene blue solution, occupying space in the gastric lumen. The Heliosphere® bag is a polyurethane-based balloon filled with 900-1,000 mL air, designed for broader gastric wall contact with a lower weight burden (35 g vs. 520 g). These structural differences influence tolerability profiles [27].

Short- and long-term weight changes with IGBs: literature comparison

IGBs promote weight loss through both mechanical and hormonal mechanisms, including enhanced gastric distension, delayed gastric emptying, and potential suppression of ghrelin secretion via fundal occupancy [28,42,43]. By occupying gastric space, balloons increase satiety signals through vagal pathways and reduce overall caloric intake, contributing to weight loss. Larger-volume balloons have been associated with greater weight loss, likely due to stronger mechanical effects and prolonged gastric retention [28,42]. However, studies report inconsistent changes in ghrelin levels following IGB placement, suggesting that additional hormonal and neural pathways contribute to their efficacy and that weight loss mechanisms are multifactorial [43].

Evidence from systematic reviews and meta-analyses confirms the short-term efficacy of IGBs, demonstrating average weight loss of 12-15 kg, equivalent to 12-18% TBWL at six months post-insertion. Notably, IGBs have been shown to achieve approximately 6.7 kg greater weight loss compared to lifestyle interventions alone, underscoring their role as a more effective initial strategy in obesity management [44]. This magnitude of weight loss, although less than that achieved with bariatric surgery, is clinically relevant, as it is associated with improvements in obesity-related comorbidities and quality of life.

Despite these benefits, the long-term durability of weight loss achieved with IGBs remains limited. Data indicate that 35-50% of patients experience weight regain within six to 12 months following balloon removal, and only 23-27% of individuals maintain greater than 20% excess weight loss (EWL) at two to five years [45]. This trend of weight regain post-device removal reflects physiological adaptations and behavioral challenges commonly observed in obesity treatment.

Notably, up to 60% of patients eventually require bariatric surgery due to weight rebound, indicating that while IGBs are effective in achieving initial weight loss, they are not definitive long-term solutions for many patients [17,41]. Nevertheless, IGBs contribute to the improvement of metabolic parameters, including reductions in fasting glucose and insulin resistance, although robust data on long-term comorbidity resolution remain limited [45].

Early weight loss following IGB placement is a significant predictor of sustained weight reduction, emphasizing the importance of patient adherence to dietary and lifestyle modifications during and after balloon therapy [3,17,46]. This highlights the role of IGBs as temporary adjuncts within a comprehensive obesity management framework, where structured maintenance programs and lifestyle support are essential for prolonging weight loss benefits. Additionally, IGBs can function as a bridging strategy to bariatric surgery in appropriate candidates, optimizing preoperative weight reduction and enhancing surgical outcomes [47].

Sustained weight loss holds critical clinical relevance; even modest long-term weight reduction significantly lowers obesity-related comorbidity risks. Specifically, maintenance of ≥5-10% weight loss is associated with substantially reduced incidence and progression of type 2 diabetes, cardiovascular events, and mortality [3].

Geographic variations in weight loss

Geographic disparities were notable, with Middle Eastern cohorts achieving 8.6% greater excess weight loss (EWL) than European patients (p = 0.02). Middle Eastern studies reported 10-17 kg short-term weight loss (equivalent to 10-19% total weight loss) and 38.5-55.6% EWL, although 34.7-78.7% of patients experienced weight regain or required additional interventions [18,19,48,49]. Low complication rates, including a 2.3% early removal rate, were observed, along with gender disparities indicating a higher %EWL in women. The superior short-term outcomes in Middle Eastern studies are likely driven by structured dietary protocols, although comprehensive long-term strategies remain lacking [19,50].

In contrast, European studies demonstrated slower weight regain, with an increase of 1.2 kg/m² at 24 months, attributed to multidisciplinary care models incorporating Mediterranean diet protocols [51]. Adherence to follow-up, rather than the type of device used, was found to strongly predict sustained weight loss (B = 0.24, p < 0.001), highlighting the critical role of post-procedural care intensity in contributing to regional differences in outcomes [52,53].

Clinical implications

BIB devices should be prioritized for their efficacy, despite higher transient nausea rates [50]. IGBs are most effective within comprehensive programs, as structured care correlates with slower regain [54]. Patient selection is critical: those with binge-eating disorders experienced 40% faster regain, underscoring the need for psychological screening. A tailored approach integrating behavioral and nutritional support is essential [55,56].

Mechanistic insights

Fluid-filled balloons enhance ghrelin suppression and vagal signaling, driving short-term weight loss. However, hormonal adaptations, such as leptin rebound, may fuel regain, mirroring post-bariatric surgery trends. Combining IGBs with GLP-1 agonists reduces weight recurrence by 40% in pilot trials, positioning IGBs as transitional tools augmented by pharmacotherapy [3,57,58].

Evolving technologies in intragastric balloon therapy

Next-generation IGBs address key limitations of traditional devices through innovative design features. Adjustable balloons (e.g., Spatz3® (Spatz FGIA Inc., Jericho, NY)) incorporate a valve system enabling in situ volume modifications (500-800 mL), permitting downsizing to mitigate intolerance (e.g., nausea) or upsizing to overcome weight-loss plateaus [59]. Procedureless systems (e.g., Elipse® (Allurion Technologies, Natick, MA)) utilize swallowable capsules that expand to 550 mL intragastrically, degrading spontaneously after four months without endoscopic intervention. Though achieving 10.7% TBWL at four months, significant attrition (24% non-responders) and limited long-term data constrain their utility [60]. Gas-filled balloons (e.g., Obalon® (Obalon Therapeutics Inc, Carlsbad, CA)) deploy ≤3 capsules inflated with 250 mL gas to minimize nausea; however, reduced volume correlates with modest efficacy (7.1% TBWL at six months) and 15% early retrieval rates [61].

Limitations

High heterogeneity (I² = 60.55%) reflects inconsistent follow-up protocols. Publication bias (p = 0.04) may inflate efficacy, though sensitivity analyses confirmed robustness. Five-year data and metabolic outcomes (e.g., diabetes improvement) were underreported, necessitating standardized metrics.

Future research directions

Key research gaps must be addressed, including the need for standardized long-term reporting metrics such as five-year percentage of TWL (%TWL) to better evaluate outcomes [62], alongside comparative trials assessing the efficacy of intragastric balloons (BIB) against newer devices like the Spatz3 adjustable balloon, etc. [59-61,63-64]. Cost-effectiveness analyses in low-resource settings are also critical, particularly where IGBs could function as a temporary intervention before definitive bariatric surgery [46].

Further investigation into combination therapies, such as integrating IGBs with GLP-1 agonists or behavioral interventions, may enhance the durability of weight loss and refine patient selection criteria, thereby optimizing personalized obesity care pathways [58]. Despite offering a less invasive option for select patients, the declining utilization of IGBs highlights evolving trends in obesity management strategies [65]. Needless to say, IGBs can play an intermediary role prior to bariatric surgery [66]. It also emphasizes the need for robust, longitudinal data to inform clinical practice and policy decisions [3,8].

## Conclusions

IGBs offer minimally invasive short-term weight loss, particularly with BIB in structured programs. The BIB exhibited superior durability to the Heliosphere device, with greater weight loss maintenance over time. While not standalone solutions, they serve as bridges to sustained management where surgery is limited. Long-term success requires lifestyle support, patient selection, and combination therapies. As obesity care evolves, IGBs remain one component of individualized strategies to achieve ideal body weight.

## Appendices

Appendix A

**Table 3:** Detailed Search Strategies Across Databases for Meta-Analysis on Intragastric Balloon and Short- and Long-Term Weight Loss and BMI Outcomes [tiab] - search in Title and Abstract fields (PubMed); [mh] - MeSH (Medical Subject Headings) term search (PubMed); exp - explode Emtree term to include the term and all narrower related terms (Embase); / - Emtree subject heading (Embase); .ti,ab,kw. - search in Title, Abstract, and Keyword fields (Embase); TS - Topic Search (searches Title, Abstract, Author Keywords, and Keywords Plus in Web of Science)

Database	Platform/Interface	Search String	Filters and Notes	Results
PubMed	Web	("intragastric balloon"[tiab] OR "gastric balloon"[tiab] OR "intragastric balloon"[mh] OR "gastric balloon"[mh]) AND ("obesity"[tiab] OR "weight loss"[tiab] OR "weight reduction"[tiab] OR "BMI"[tiab] OR "body mass index"[tiab] OR "obesity"[mh]) AND ("long-term"[tiab] OR "long term"[tiab] OR "follow-up"[tiab] OR "long-term follow-up"[tiab] OR "weight maintenance"[tiab])	Date: 2000/01/01-2023/12/31; Syntax: [tiab] = title/abstract; [mh] = MeSHFilters: Humans	-
Embase	Ovid	1. exp intragastric balloon/ or gastric balloon/ 2. (intragastric balloon OR gastric balloon).ti,ab,kw. 3. 1 OR 2 4. exp obesity/ OR exp weight reduction/ OR exp body mass index/ 5. (obesity OR "weight loss" OR "weight reduction" OR BMI OR "body mass index").ti,ab,kw. 6. 4 OR 5 7. ("long-term" OR "long term" OR "follow-up" OR "weight maintenance").ti,ab,kw. 8. 3 AND 6 AND 7	Date: 2000-2023; Syntax: .ti,ab,kw. = title/abstract/keyword; Filters: Humans, exclude conferences	-
Cochrane Library	Wiley	([mh "intragastric balloon"] OR "intragastric balloon" OR "gastric balloon") AND ([mh "obesity"] OR "obesity" OR "weight loss" OR "weight reduction" OR "body mass index" OR BMI) AND ("long-term" OR "long term" OR "follow-up" OR "weight maintenance") in Title, Abstract, Keyword	Date: 2000-2023; Scope: Trials, reviews, CENTRAL Register	-
Web of Science	Web of Science	TS=(("intragastric balloon" OR "gastric balloon") AND ("obesity" OR "weight loss" OR "weight reduction" OR "BMI" OR "body mass index") AND ("long-term" OR "long term" OR "follow-up" OR "weight maintenance"))	Date: 2000-2023; Indexes: SCI-EXPANDED, SSCI, AHCI, ESCI; Document Types: Article, review	-
Manual Search	-	Screening reference lists of included studies and relevant systematic reviews	No date restrictions	-
Update (All DBs)	-	Identical strings re-executed on June 1, 2024 (Date filter: 2023-2024 to capture new records post-initial search)	Inclusion: Added 2023-2024 records missed initially	27 included

Appendix B

**Table 4:** Appraisal of Methodological Quality Using the MINORS Tool for Studies Investigating Intragastric Balloons in Obesity Management (N = 27) MINORS criteria scored as 0 (not reported), 1 (reported but inadequate), and 2 (reported and adequate). Maximum score = 24 (16 for non-comparative studies). References are in the square brackets. MINORS - Methodological Index for Non-Randomized Studies

Author	Aim	Consecutive	Prospective	Endpoints	Unbiased	Follow-Up	Loss < 5%	Size Calculation	Control	Contemporary	Baseline	Statistics	Total
Kotzampassi et al. [17]	2	2	2	2	2	2	2	2	0	0	0	0	16
Al Kahtani et al. [18]	2	2	2	2	2	2	2	2	2	2	1	2	23
Almeghaiseeb et al. [19]	2	2	2	2	2	2	2	2	0	0	0	2	18
Tai et al. [20]	2	2	0	2	2	2	2	2	0	0	0	2	16
Farina et al. [21]	2	2	2	2	2	2	2	2	0	0	0	0	16
Giuricin et al. [22]	2	2	2	2	2	2	1	2	0	0	0	0	15
Nunes et al. [24]	2	2	0	2	2	2	0	0	0	0	0	0	10
Fuller et al. [25]	2	2	0	2	2	2	1	0	0	0	0	0	11
Genco et al. [26]	2	2	2	2	2	2	2	2	2	2	1	2	23
De Castro et al. [27]	2	2	2	2	2	2	2	2	2	2	1	2	23
Sallet et al. [28]	2	2	2	2	2	2	2	2	0	0	0	0	16
Gümürdülü et al. [29]	2	2	2	2	2	2	1	2	0	0	0	0	15
Genco et al. [30]	2	2	2	2	2	2	1	2	0	0	0	0	15
Alfredo et al. [31]	2	2	0	2	2	2	0	0	0	0	0	0	10
Palmisano et al. [32]	2	2	2	2	2	2	0	2	0	0	0	2	16
Courcoulas et al. [33]	2	2	2	2	2	2	2	2	0	0	0	0	16
Crea et al. [34]	2	2	2	2	2	2	2	2	0	0	0	0	16
Dogan et al. [35]	2	2	2	2	2	2	2	2	0	0	0	2	18
Mitura et al. [36]	2	2	2	2	2	2	2	2	0	0	0	2	18
Ribeiro da Silva Silva et al. [37]	2	2	2	2	2	2	1	2	2	2	1	2	22
Escudero Sanchis et al. [38]	2	2	0	2	2	2	0	0	0	0	0	0	10
Mitura et al. [39]	2	2	2	2	2	2	0	2	2	2	2	0	20
Herve et al. [40]	2	2	2	2	2	2	1	2	0	0	0	0	15
Dastis et al. [41]	2	2	2	2	2	2	2	1	0	0	0	0	15
Mathus-Vliegen et al. [43]	2	2	2	2	2	2	2	2	0	0	0	0	16
Genco et al. [52]	2	2	2	2	2	2	1	2	0	0	0	0	15
Melissas et al. [66]	2	2	2	2	2	2	2	2	0	0	0	0	16
